# Woman at Home

**Published:** 1887-11-05

**Authors:** 


### THE DOCTOR'S PULPIT.

### WOMAN AT HOME.

Perfect health is like fine, ripe fruit. The fruit as a finished product is independent, and complete in itself; but it presupposes buds and leaves, branches and trunk, roots and soil, sun, rain, and atmosphere. So health is dependent on a thousand influences, of which one of the most significant is that assembly of circumstances which makes up the complex idea of "home." The ancient philosopher who wished to understand man took into consideration everything which had a bearing upon human life; he considered nothing foreign to his study which belonged to man; as the moderns say, he studied the individual, together with his environments. Without such a broad range of study, man as a reasoning creature could by no means be understood. We must do the same with health. Let no foolish pedant charge the doctor with wandering from his text because he travels beyond the range of pill and physic bottle, stethoscope and thermometer, scalpel and microscope. Everything that nearly or remotely affects a man's well-being is the proper concern of the doctor. It has often been stated in these pages, and is here reaffirmed, that man is one—not two. The whole man includes what, in generally understood phraseology, is called body, soul, and spirit. He who treats man as a mere physical organism, taking no account of his mental and spiritual parts, is narrower than his subject; he who looks exclusively to the mental and spiritual is also narrower than his subject. The true physician tries to look on all sides; and he only succeeds in understanding man by studying the individual as well as the race, the race as well as the individual. There is a sense in which one man is the key to all men; there is also a sense in which all men form the only possible key to any man. "Not this man nor that man, but *all* men, make up mankind."

For most men life is made up of work and home—business and domestic concerns. Man is, as a rule, what his business makes him. He is a farmer, or a sailor, or a physician. It is seldom that he is of such a cosmopolitan mould as to be simply a man. Woman also is a specialist, and her duties for the most part lie at home. Whether for weal or for woe, home is woman's kingdom. Many women are tempted to rebel against this arrangement, and many others are separated from their homes by the force of circumstances, and have to enter the lists with men in professions and other callings. But these are the exceptions. The rule still is that woman plays her part as chief manager of the domestic circle.

But now it must be considered that domestic concerns constitute at least one-half of life for ordinary families. There is a real and most important partnership between husband and wife in the conduct of life's business. If a moment's reflection be given to the question it is easily seen that the trade or profession by which a man lives is only a part of the scheme of his life. He fails or he succeeds, not merely by his prosperity or failure in business, but by his progress in business added to his home management. In the olden times, when a man did not live in a villa, but over his shop, it used to be said that he must ask his wife whether he was to "live or not." The connection of the wife and family with the business does not appear to be so intimate now, and especially in large towns. But it is as intimate in reality, perhaps even more so, because where there are two establishments to maintain instead of one it becomes of prime importance that both of them be equally well managed. It is often a difficult thing to get a capable "head" for one establishment. The difficulty is doubled when two capable heads are required.

Married women should take this view of their own position. They should not think that they are put into inferior places by being made managers of the domestic branch of the copartnery. They should rather look with pride upon the equality which is thus assigned to them, and resolve that their branch shall be at least as well managed as the branch in the city or elsewhere. But how does the matter really stand in actual life? We will go back a little upon the early days of men and of women. A youth who is intended for a definite business, be it what it may, is always trained for it. The training may be good, or it may be bad, or it may be indifferent; but a positive and purposed training there always is. A certain end is in view, and means are taken to accomplish it. The lawyer, the doctor, the clergyman, the tailor, the builder, even the statesman, are all put through a course of instruction; and nobody thinks of employing them unless they can furnish evidence of a certain degree of competency. How stands the case with young women? What proportion of them are taught to regard domestic management as a business? How many of them receive any systematic training for the conduct of a household? It is at least as difficult to manage a house well as to manage a grocer's shop; at least as much skill is required in the domestic head of the family as in the post-office or telegraph clerk. Yet the grocer gives six or seven years to the study of his business before he ventures on his own account; and the telegraph clerk can obtain no employment except on passing a qualifying examination. We do not desire to use strong language, or to declaim; but we do say that looking at the subject with the least practical eye, it is nothing less than astonishing that things should be as they are. That men should have so long endured it says much for their good nature as well as for their stupidity. That women should have been, and should still remain, content with it, is a stronger argument in proof of their decided mental inferiority than volumes of logic or scores of university successes can avail to disprove or to weaken in the slightest degree. "By their fruits ye shall know them." That is a test capable of universal application, and of absolutely unerring value. Tried by this test, where do women stand? Exactly here: that not one in a hundred of them thinks it the least necessary to have any special training for the performance of the duties of life. We are not deriding them; far, very far from it. On the contrary to practical men and women, the fact is humiliating, disheartening, terrible. Most of us see a tremendous struggle awaiting the civilized races. We see a daily increasing necessity for all the mental, physical, and moral strength of which we are capable; and along with that we see women as a class of skilled domestic managers running absolutely to waste; and as the result of their incapacity not only failing to add to the resources of the race, but drawing upon the resources of men to such an extent as seriously to imperil their chances in the struggle.

We speak from the standpoint of the man and the doctor. No one sees so well as the thinking physician the strain upon the men of the present generation; and, as a member of the commonwealth, he cannot help looking round for the means of diminishing it. In arriving at his diagnosis he is bound to take causes into consideration. What are the causes of the pressure to which men in the large towns of Great Britain are now subjected? One of the principal is this: The necessity for making a comparatively large income in order to maintain family life at the level to which it has attained, and at which it seems necessary it should be maintained, if there is to be no falling behind in the race. If the same relative position could be preserved on a considerably smaller income, the strain on the resources of men would be diminished, and the injurious pressure upon their health and life forces would also be correspondingly lessened. Is there any available method of making a smaller income serve the purpose of the present one? There is! Increase the competency and skill of women as domestic managers, and the thing is done. We make bold to express the conviction that a thoroughly trained and efficient class of housewives would give all the practical comforts, pleasures, and advantages secured to the family by the present race of housewives, at twothirds the cost. That is much the same as saying that if all housewives were competent, the burden of life would be so much lightened for men as to make it comparatively easy and pleasant to bear. As matters are at present, most men have to do the work of one man and an appreciable fraction of another; and hence to them there is bound to result early disablement and premature old age. These statements are made with the view of calling the attention of clever and capable women to a subject which is at least as important as the Irish or the Disestablishment question. It is believed that large reforms are possible; that the bulk of women have the desire for such reforms, and that what is wanted is that the facts shall be clearly set forth, and that the able and competent among the gentler sex shall take the matter up in a practical and determined spirit. It is proposed to return to the subject, and to let women know, from a man and a doctor's point of view, at least what reforms are necessary. It may then be found possible to make life healthy, and perhaps also more or less happy.

